# Intraretinal Microvascular Abnormalities and Venous Beading Have Different Genetic Profiles in Caucasian Patients with Non-Proliferative Diabetic Retinopathy

**DOI:** 10.3390/vision7010018

**Published:** 2023-03-02

**Authors:** Elizabeth Pearce, Sobha Sivaprasad, Suzanne Broadgate, Christine Kiire, Susan M. Downes, Stephanie Halford, Victor Chong

**Affiliations:** 1 King’s College Hospital NHS Trust, London SE5 9RS, UK; 2 UCL Institute of Ophthalmology, London EC1V 9EL, UK; 3 NIHR Moorfields Biomedical Research Centre, Moorfields Eye Hospital, London EC1V 2PD, UK; 4 Nuffield Laboratory of Ophthalmology, Nuffield Department of Clinical Neuroscience, University of Oxford, Oxford OX3 9DU, UK; 5 Oxford Eye Hospital, John Radcliffe Hospital, Oxford University Hospitals, NHS Foundation Trust, Oxford OX3 9DU, UK

**Keywords:** non-proliferative diabetic retinopathy, intraretinal microvascular abnormalities, venous beading, single nucleotide polymorphisms

## Abstract

Diabetic Retinopathy (DR) is a leading cause of preventable visual impairment in the working age population. Despite the increasing prevalence of DR, there remain gaps in our understanding of its pathophysiology. This is a prospective case-control study comparing the genetic profiles of patients with no DR vs. non-proliferative DR (NPDR) focusing on intraretinal microvascular abnormalities (IRMA) and venous beading (VB) in Caucasians. A total of 596 participants were recruited to the study; 199 with moderate/severe NPDR and 397 with diabetes for at least 5 years without DR. Sixty-four patients were excluded due to technical issues. In total, 532 were analysed; 181 and 351 were in the NPDR group and no DR group, respectively. Those with severe IRMA and VB had distinctly different genetic profiles from each other and from the no DR group, which further supports the theory that these two features of DR might have different etiologies. This also suggests that IRMA and VB are independent risk factors for the development of PDR and may have different pathophysiologies. If these findings are confirmed in larger studies, this could pave the way for personalised treatment options for those more at risk of developing different features of NPDR.

## 1. Introduction

Diabetic Retinopathy (DR) is a leading cause of preventable visual impairment in the working age population, with recent rising prevalence observed in elderly people due to increasing longevity [1,2,3]. Despite a significant body of research in DR, there remain gaps in our understanding of its pathophysiology.

Known risk factors such as poor glycaemic control and duration of diabetes [4] do not explain why some individuals are more prone to sight-threatening DR (STDR). In addition, DR often manifests gradually, over a period of 10 years, and progresses from no retinopathy to proliferative diabetic retinopathy (PDR). The speed of progression through the severity levels of DR varies enormously. Subjects with many years of poor diabetic control may never develop STDR, but in some with good control, severe diabetic eye disease can develop rapidly [5]. This suggests that other risk factors need to be investigated to fully explain the onset and progression of DR. Therefore, we hypothesize that genetic predisposition may also contribute to the different vascular phenotypes of DR. Discovering genetic associations of DR may also help in stratifying patients’ appointment intervals based on these risks, thereby more efficiently utilizing resources. Identifying candidate genes involved in the pathogenesis of DR may also help develop targeted therapeutic strategies [6] and inform investigations of future drug targets.

Studies have found that DR is driven by multiple genes [7]. Twin studies have described a strong link between retinopathy levels in Type 2 diabetes mellitus (T2DM), but not so in twins with Type 1 diabetes mellitus (T1DM), suggesting that genetic factors may determine the extent and severity of DR in T2DM, but environmental factors may have more influence on DR in T1DM [8]. In this twin study, 35 of 37 sets of twins with T2DM, with similar durations of DM, shared the same DR grade, whereas, of the 31 sets of T1DM twins, 10 had markedly differing DR severities, despite each of the sets of twins also having very similar durations of DM. Most twins with T2DM had been living apart throughout the duration of their diabetes, implying that environmental factors are less likely to be responsible for the similarities and adding weight to the argument that DR in T2DM may be genetically driven.

Moderate/severe non-proliferative DR (NPDR) is characterized by the presence of three features: deep haemorrhages (DH), venous beading (VB), and intraretinal microvascular abnormalities (IRMA). VB and IRMA are considered important risk factors for the progression to PDR and subsequent visual loss [9]. Despite being distinct anatomical features, VB and IRMA are grouped together as moderate to severe NPDR with a significant risk of progression to PDR. We have previously shown that VB does not respond to anti-vascular growth factor (anti-VEGF) inhibitor agents, unlike IRMA, suggesting that they may be driven by different mechanistic pathways [10]. It remains unclear whether they have the same etiology or pathophysiology and are induced by the same trigger such as ischaemia.

Very few studies have examined the individual risk of the progression of VB and IRMA since the landmark trials of the 1980s (Diabetic Retinopathy Study (DRS), and The Early Treatment of Diabetic Retinopathy Study (ETDRS)). A more detailed characterization of these three features may improve the prediction of visual prognosis and help us better understand the pathophysiology. In addition, the genetic profile of DR has previously been studied without investigating the genetic risk factors for these individual lesions. In this study, the differences between no DR and NPDR were examined. However, the focus was VB and IRMA, as DH is very common in moderate and severe NPDR. The study aimed to identify, if any, genetic differences between subjects with these features.

This is a novel approach. Previous studies have looked at genetic associations in subjects with DR vs. no DR. We used a candidate gene approach to increase the likelihood of identifying differences between our targeted features (VB and IRMA) within the DR disease class.

## 2. Materials and Methods

The study was granted approval by the South West–Cornwall and Plymouth Research Ethics Committee (Research Ethics Approval Number: 10/H0203/14), the Health Research Authority and Trust management of Oxford University Hospitals NHS Trust, and King’s College Hospital NHS Trust, and adhered to the tenets of the Declaration of Helsinki. Informed consent was obtained from all participants.

This was a prospective case-control study, involving Caucasians with T1DM and T2DM. Previously, our group performed a search of the literature of studies examining genetic associations with diabetic macular edema (DME) and T2DM [11]. This was enhanced by searching for studies of genetic associations for DR, in particular, NPDR. The US National Library of Medicine Institute of Health’s PubMed.gov search engine was used, entering the terms ‘non-proliferative diabetic retinopathy’, ‘genetics’, and/or ‘SNPs’, between 2016 and 2019. Approximately 100 single nucleotide polymorphisms (SNPs) of interest were identified. The literature review identified a number of studies that reported positive associations between a variety of SNPs and DR when DR was compared to no DR or NPDR vs. PDR. Reports show conflicting results because of different study populations, varying sample sizes, and different endpoints [12]. We selected 28 SNPs involved in vascular pathology which are likely to contribute to the development of VB and IRMA.

### 2.1. Study Participants

Subjects were recruited from retinal clinics and diabetic retinopathy screening clinics at King’s College Hospital NHS Foundation Trust and Oxford Eye Hospital, Oxford University Hospitals NHS Foundation Trust. Caucasian subjects with T1DM or T2DM aged 18 or over and able to give informed consent were invited to join the study. Those recruited to the no DR group were required to have been diagnosed with diabetes at least 5 years prior to enrollment. Enrollment was between 2017 and 2019.

### 2.2. Study Assessments

The study required one visit, and all data were collected during the subject’s scheduled regular appointment. Those recruited to the no DR group of the study had fundus photography as part of their routine care. After mydriasis, two images, one centered on the optic disc and one centered on the macula, were taken with a digital fundus camera (Topcon Non-Mydriatic Retinal Camera TRC-NW8, Tokyo, Japan). These images were examined for signs of DR. If they met all the inclusion criteria (diagnosis of DM at least 5 years previously, aged 18 years or older, no DR), they were recruited to the control arm of the study.

Those recruited to the NPDR group of the study had ultrawide field scanning laser ophthalmoscope (UWFSLO) (Optomap 200Tx; Optos plc, Dunfermline, UK) Optomap images taken after mydriasis as part of the patient’s routine care. All patients who met the inclusion criteria (aged 18 years or older and a diagnosis of NPDR) and were willing to participate, were recruited as cases to the study. Exclusion criteria: previous pan-retinal photocoagulation (PRP) laser and prior intravitreal anti-VEGF injections or intravitreal steroid injections.

A short questionnaire was administered. This recorded the number of years’ duration of DM; whether T1DM or T2DM; and whether there was a history of angina, stroke, renal disease, or laser treatment for diabetic eye disease. Current medication and smoking status were recorded, and the participants’ most recent recorded glycated hemoglobin level (HbA1c) was obtained from their clinical notes.

A buccal cell sample was taken from the inside of the mouth of each participant with a buccal collection brush and stored in a tube of Cell Lysis Solution (Qiagen, Manchester UK) as per the standard operating procedure. The samples were purified using the Gentra Puregene Buccal Cell Kit, Qiagen, Manchester, UK. In this cohort, a ratio of 1.6–2.1 at A260/280 was deemed acceptable to be used for further analysis.

The Oxford Genomics Centre, Wellcome Trust’s Infinium Global Screening 24v2.0x bead-chips was selected as it covered many genes and included most of the SNPs of interest and was most appropriate for this study. The DNA samples from 199 patients with NPDR and 397 diabetic patients with no DR were collected. The case-control comparisons were between those with moderate–severe NPDR and no DR; those with VB and no DR, and those with IRMA and no DR. The level of VB and IRMA included were both Grade 3 of the ETDRS classification [13].

**Figure 1:**
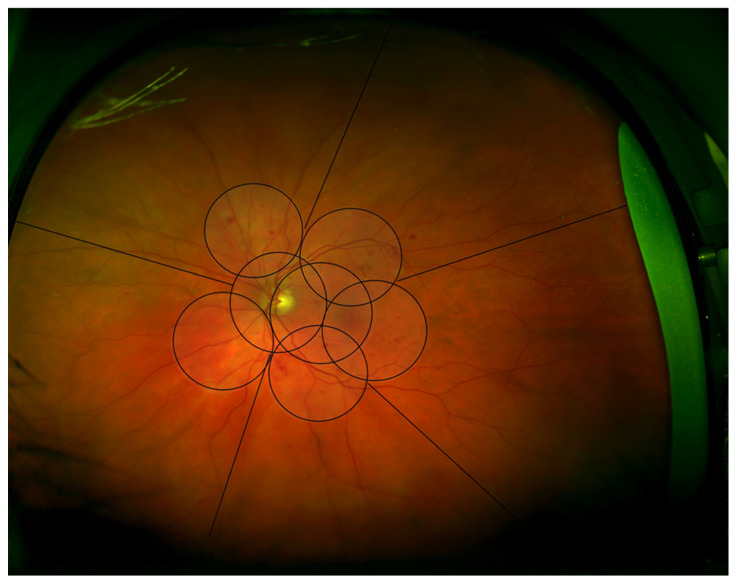
Optomap Image illustrating the areas graded, the central seven fields divided into quadrants centered on the optic disc, and five peripheral areas.

The Early Treatment Diabetic Retinopathy Study (ETDRS) used seven standard field 35 mm film stereoscopic color photographs, each image being 30° to 35° wide, to assess DR severity levels. Field 1M is centered on the temporal edge of the optic nerve, field 2 on the macula, and field 3M on the temporal macula. The four outer fields image the superior temporal, inferior temporal, superior nasal, and inferior temporal fundus. A seven-field overlay, equivalent to ETDRS 30-degree seven standard field 35mm stereoscopic color photographs, was superimposed on the Optomap image using Adobe Photoshop CS2 (Adobe Systems Incorporated, San Jose, CA, USA). The two central ETDRS fields covered the macula and optic disc. This enabled the ETDRS seven-field area of the posterior pole to be assessed (Figure 1).

The images were graded according to the ETDRS diabetic retinopathy severity scale (DRSS) grading system. The seven standard ETDRS fields were graded by quadrants centered on the optic disc; superior temporal, superior nasal, inferior nasal, and inferior temporal (Figure 1). This method has previously been described by this group, based in Joslin Diabetes Centre, Boston [14,15].

The grading quadrants were then extended to the periphery, using the same quadrants (superior, inferior, temporal, and nasal) as well as an additional middle temporal area (Figure 1). Each quadrant was evaluated for the presence and severity of the three features of NPDR: deep haemorrhages (DH), venous beading (VB), and intraretinal microvascular abnormalities (IRMA).

A grading audit was performed. A sample of 10% of images was re-graded by the primary grader, a sample of 10% of images was graded by another experienced grader, and the results compared.

Intra-grader agreement was found to be 87.5% within one DRSS step and 100% within two DRSS steps. Inter-grader agreement was found to be 85% within one DRSS and 100% within two DRSS steps. When differences were found, cases were discussed and consensus was sought.

## 3. Results

In total, 596 participants were recruited to the study, including 199 with moderate/severe NPDR (NPDR group) and 397 with diabetes for at least 5 years but no DR (no DR group). Twenty samples failed to yield enough DNA to be analysed, leaving 576 samples that were sent to the Wellcome Centre Human Genetics, University of Oxford to be analysed. Of these, 44 were removed from the analysis as they failed to yield meaningful data. In total, 532 samples were analysed, of which 181 and 351 were in the NPDR and no DR groups, respectively.

The demographic and features analysis included the 532 participants whose samples could be analysed and are displayed in Table 1.

The no DR group was significantly older, *p* < 0.0001; had a lower HbA1c, *p* = 0.0003; and a shorter duration of diagnosed DM, *p* = 0.0049. As participants in the control group might develop DR in later life (and were older at the time of analysis), the findings are likely to become more significant, hence the analysis was not adjusted for age. There was no difference between sex for cases and controls (*p* = 0.834, Chi-Square = 0.044). In both groups, T2DM was much more prevalent (no DR T2DM *n* = 340, T1DM *n* = 10; and NPDR T2DM *n* = 153, T1DM *n* = 25). Information about the type of DM was not available for four subjects. When looking at the prevalence of DR among those with T1DM and T2DM, those with T1DM were more likely to have DR than those with T2DM, *p* < 0.00001, Chi Square = 23.86. Analysis of the NPDR group revealed the mean age of those with IRMA showed a trend to be lower than those without (IRMA mean 56.9 years, without IRMA 60.94, *p* = 0.059). Those with IRMA had a slightly longer duration of DM, IRMA mean of 21.30 years, and without 16.26 years (*p* = 0.309) and slightly lower HBA1c, IRMA mean 8.53, and without 8.81 (*p* = 0.378). The findings for VB were similar to those with IRMA in terms of age; patients were slightly younger, but the duration of DM was shorter, and HBA1c was slightly higher (the opposite of IRMA), although these results were not statistically significant. In Table 2, the results of the Illumina analysis are displayed.

The TGF-b1 SNP rs1800470 showed the highest correlation (*p* = 0.015) for the group NPDR vs. no DR, followed by the SLMAP SNP rs17058639 (*p* = 0.035) and CFB rs1048709 (*p* = 0.042). None of the SNPs selected showed any association with those with VB. Four of the VEGFA SNPs, the Rho GTPase activating protein 22 (ARHGAP22) SNP rs4838605, and the TGF-b1 SNP (rs1800470) (also associated with NPDR) showed statistically significant associations with the presence of IRMA. CFH, C5, and VDR showed no significant associations with DR, VB, or IRMA.

**Figure 2:**
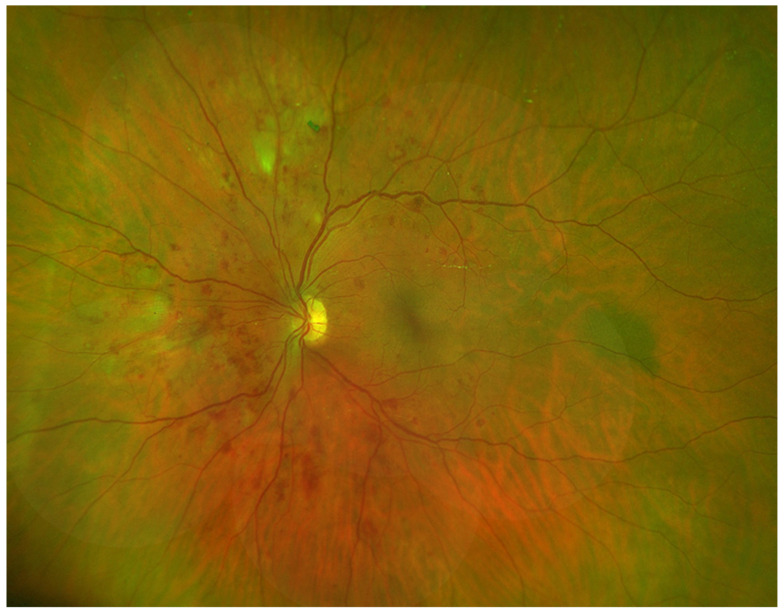
Optomap Image of Participant with DH, VB, and IRMA.

Figure 2, shows an Optomap image of a participant with the three features of NPDR: DH, VB and IRMA.

## 4. Discussion

This prospective case-control study comparing the genetic profiles of patients with no DR vs. NPDR showed that there are distinctly different genetic profiles when comparing diabetic patients with and without DR, and with VB and IRMA compared to each other. The results displayed are not corrected for multiple testing. For statistical significance, a nominal *p*-value of < or = 0.0031 would have to be met if this were the case, as 17 SNPs were analysed. No SNP reached this level of significance.

We focused on three areas of interest: (a) vascular endothelial growth factor (VEGF); (b) ischaemia-mediated growth factors and cytokines, and (c) the complement pathway. These three groups were chosen because anti-VEGF is used as a therapy for diabetic retinopathy and is therefore likely to be relevant to the development of DH, VB, and IRMA. Cytokines and growth factors associated with ischaemia may also be of interest as both VB and IRMA are believed to be driven by ischaemia. These include HFE, SLMAP, VDR, TGF-b1, and ARHGAP22. A complement factor was selected as it has been implicated in other retinal vascular conditions. Seven SNPs of interest could not be examined as they were not on the Illumina Array. Another five SNPs were excluded due to a high frequency in the general population making statistical analysis impossible.

An examination of the demographic data of both groups found that those with NPDR were likely to be younger. As the NDPR group had a longer duration of DM, it is reasonable to suggest that the no DR participants with the significant alleles may develop NPDR in the future with the increasing duration of DM. By including patients in whom the duration of DM was more than 20 years as opposed to 5 years in this study, this hypothesis could be confirmed.

An analysis of the NPDR group showed no significant association with age, sex, duration of DM, or HbA1c levels when looking at the presence or absence of VB or severe IRMA. However, it is interesting that patients with VB (16.02 years) have shorter durations of DM as compared with those with IRMA (21.30 years) despite being very similar in age at 56.70 years for VB and 56.90 years for IRMA. This may suggest that VB develops first and then progresses to PDR without developing IRMA. Indeed, this theory would be consistent with the observation that VB is rarely observed alone, while severe IRMA may occur with or without VB.

### 4.1. NPDR vs. No DR Results

#### 4.1.1. Transforming Growth Factor Beta TGF-b1, rs1800470 *p* = 0.015

The strongest association in the comparison between NPDR and no DR was found with SNP TGF-b1 rs1800470. The *p*-value of this was 0.015. The result ties in with the findings that TGF-b1 has a role in angiogenesis, maintaining retinal capillaries, and is involved in cellular proliferation and differentiation, its inhibition leading to the breakdown of the blood–retina-barrier (BRB) and impaired retinal perfusion [16]. Previous studies found that TGFb signaling was important for maintaining pericyte function [16]. The loss of pericytes in the basement membrane of retinal vessels appears early on in DR and may contribute to vascular leakage, haemorrhage, and microaneurysm development [17,18]. Our results support similar findings in many other studies [19,20,21].

#### 4.1.2. Sarcolemma Membrane Associated Protein SLMAP, rs17058639 *p* = 0.035

SLMAP was also positively associated with NPDR vs. no DR in our study. SLMAP has a role in vascular endothelial dysfunction and may be a marker of microvascular dysfunction in diabetes. A previous study found this SNP to be associated with a higher risk of DR [22]. Vascular endothelial cell death is widespread in DR and leads to hypoxia and capillary closure [18]. The only weak genetic association with VB is with SLMAP. The result may be a coincidence, but SLMAP may play a role in vascular endothelial cell dysfunction, and its association with the dysfunction of the contraction regulation of the small mesenteric arteries in db/db mouse (a genetic diabetic mouse model) [23] is interesting. It is possible that SLAMP may be involved with the contraction regulation of retinal venules leading to venous beading.

#### 4.1.3. Complement Factor B CFB, rs1048709 *p* = 0.042

The role of complement in the development of DR is not completely understood. The complement system helps regulate the immune response and inflammation, and its dysfunction is linked to autoimmune disease [24]. DM affects the production of complement proteins, and elevated levels of CFB have been detected in the vitreous of patients with PDR, despite also being implicated in the early stages of DR [25]. Evidence from previous studies suggests the complement system is involved in the development of DR. The SNP rs1048709 was previously found to have an association with DR versus no DR in a Chinese population [26] and had a significant association with the presence of DR compared to no DR in our study. Our findings, therefore, strengthen the case for CFB’s role in the development of DR.

#### 4.1.4. Vascular Endothelial Growth Factor VEGFA, rs2010963 *p* = 0.083, ARHGAP22 rs4838605 *p* = 0.065

One VEGF SNP showed a positive association with NPDR, as well as an ARHGAP22 SNP, although these were not statistically significant (*p* = 0.083 and 0.065, respectively).

### 4.2. VB vs. No DR Results

No association was found between any SNPs studied and the presence of VB. SLMAP rs17058639 was not significant but showed a trend association, *p* = 0.106. SLAMP was found to show a positive association with the development of NPDR vs. no DR (*p* = 0.035) but not with IRMA. This finding may be explained by SLMAP being positively associated with DH, but as DH was so common in the NPDR group, its genetic associations were not studied separately. SLMAP has been implicated in vascular endothelial dysfunction in diabetes, potentially leading to DH. Furthermore, if confirmed in future studies of its importance in VB, the pathophysiology of VB may be related to vessel wall damage secondary to endothelial dysfunction. Nonetheless, most interestingly, none of the ischemic-driven genes showed any significance. This may be due to the relatively small number of patients with VB; however, significant associations were shown with IRMA with a similar number of patients.

### 4.3. IRMA vs. No DR Results

Four of the VEGF SNPS; rs2010963 *p* = 0.09, rs699947 *p* = 0.046, rs13207351 *p* = 0.035, and rs1570360 *p* = 0.05 showed a correlation with IRMA compared to those without. There was a weaker association with one VEGF SNP and NPDR vs. no DR. As previously described, VEGF has an important role in retinal angiogenesis and endothelial cell permeability and is activated by microvascular changes resulting from elevated blood glucose levels and hypoxia [6]. As previously shown by this group [10], IRMA improves after anti-VEGF therapy, so these associations are not unexpected. Numerous studies have found positive associations with DR and SNPs associated with VEGF, as discussed previously; indeed, VEGF has the greatest number of SNPs reportedly associated with DR [27].

TGFb-1 rs1800470 also showed a positive association with IRMA compared to those without, *p* = 0.02, suggesting it has a role in the development of DH and IRMA.

Rho GTPase activating protein 22 (ARHGAP22) rs4838605 (*p* = 0.048) was found to be significant in the group with IRMA compared to no IRMA. The ARHGAP22 may be involved in endothelial cell migration and angiogenesis, specifically capillary tube formation, and thereby influence IRMA development.

Previous studies have shown an association between DR and VEGF [28,29,30], as well as with NPDR and TGFb1 [19,31]; thus, our results confirming this are not surprising. Less prevalent was an association with DR and CFB, ARHGAP22, and SLAMP in previous studies.

In a complex disease such as NPDR, there are several systemic factors that can modify the disease. Additionally, some patients may have already progressed to PDR and, therefore, are not represented in the NPDR group who may also have the genetic association, thereby explaining why the results were not highly significant. Nonetheless, the differences in association with VEGF and TGFb-1 polymorphisms, and to a lesser extent CFB and ARHGAP22, between NPDR and no DR may be driven by the presence of IRMA, for which several VEGF SNPs, TGFb-1, and ARHGAP22 SNPs showed a positive correlation with IRMA’s presence. Conversely, we found no gene to be associated with the presence of VB.

These findings of VEGF, TGFb-1, and ARHGAP22 SNPs’ positive association with those with IRMA tie in with the hypothesis that IRMA may be a precursor of new vessels as they all play a role in angiogenesis, cellular proliferation, and differentiation. The pathophysiology of VB may be different and may be more related to endothelial damage in the vessel wall.

The diabetic retinopathy severity scale (DRSS) was developed based on the findings of the ETDRS. Using mathematical modelling, DH, VB, and IRMA were found to be independent risk factors for the progression from NPDR to PDR and eventually visual loss [9]. There is a degree of inconsistency in this grading system; on one hand, it was claimed that VB is the most significant risk factor for progression to PDR [9,11]. Yet, to be graded as severe NPDR (DRSS 53), an eye requires two quadrants of VB. However, if the eye has only one quadrant of severe IRMA, it is also severe NPDR (DRSS 53), that is, considered an equivalent risk factor for progression. This inconsistency is reflected further down the scale at DRSS 47. At this level, VB in one quadrant has a score of 47, yet four quadrants of mild IRMA (less than standard photograph 8A) are required to be DRSS 47. Despite being identified as independent risk factors, they often coexist; for instance, VB alone without DH and IRMA are rare. It is claimed that both VB and IRMA are secondary to ischaemia [32,33] and related to vascular closure [34] and pericyte loss [35]. In this study, we aimed to better understand these features in patients with DRSS 43 to 53 (moderate/severe NPDR). With similar numbers of patients with severe IRMA and VB, we found distinctly different genetic profiles. Genes that are well known to be related to ischaemia showed positive associations with IRMA but not with VB (unadjusted *p*-values). The association with IRMA may not be highly significant due to the low number of study participants; however, the differences between the results for the group with IRMA and the group with VB were clear.

### 4.4. Limitations of the Study

The study size was relatively small, and the SNPs selected may be biased towards the authors’ research interests. Nonetheless, the SNPs were pre-selected and the two focused features, VB and IRMA, showed clear differences in their genetic profile relative to no DR and to each other, despite a slight difference in the numbers in each group (79 with IRMA, 50 with VB). The no DR arm was not imaged with UWFSLO but with two color fundus images, as this is standard of care. Peripheral lesions may therefore have been undetected in this group; nonetheless, the grading of VB and IRMA was based on the central seven fields, so it is comparable. We did not correct for age, duration of diabetes, and diabetic control, the most important risk factors for DR [4,36,37,38,39]. In the VB and IRMA groups, there was no difference in age or HbA1c levels, and a small difference in the duration of diabetes. As this study was designed to identify differences between VB and IRMA and no meaningful differences were found, no correction for these variables was therefore made. None of these co-morbidities would likely change the results, as we were comparing IRMA and VB in patients who already have NPDR.

## 5. Conclusions

The different genetic findings for those developing VB and IRMA further strengthen the theory that these two features of DR have different etiologies. If these findings are confirmed in larger studies, this could pave the way for personalized treatment options for those more at risk of developing different features of NPDR. To date, little is understood about the etiology of VB and IRMA. Both are frequently found adjacent to areas of ischaemia, suggesting ischaemia’ s role in their etiology. This study suggests that they might be considered independent risk factors for the development of PDR and behave differently in terms of genetic origin in addition to their differing responses to anti-VEGF treatment. This study adds further weight to the different pathophysiologies of VB and IRMA, further suggesting that IRMA is ischaemia driven. What drives the formation of VB remains unclear.

## Figures and Tables

**Table 1:** Demographic data of those with genetic analysis.

	No DR (*n* = 351)	NPDR (*n* = 181)	VB (*n* = 50)	IRMA (*n* = 79)
Mean Age (years) (SD)	66.18 (14.2)	59.19 (13.2)	56.7 (12.6)	56.9 (12.9)
Sex (Male)	57.8%	60.5%	55.2%	53%
HbA1c (SD)	7.65 (1.58)	8.68 (1.26)	8.94 (1.9)	8.53 (1.72)
Mean duration of DM (years) SD	12.15 (6.77)	18.49 (8.03)	16.02 (7.76)	21.3 (8.22)

**Table 2:** Results of Illumina Analysis with un-adjusted *p*-value.

Gene	SNP	NPDR vs. No DR NPDR (*n* = 181) No DR (*n* = 351)	VB vs. No DR VB (*n* = 50) No DR (*n* = 351)	IRMA vs. No DR IRMA (*n* = 79) No DR (*n* = 351)
CFB	rs1048709	0.042	0.199	0.173
CFH	rs800292	0.36	0.228	0.669
C5	rs17611	0.501	0.557	0.36
VEGFA	rs2010963	0.083	0.487	0.09
VEGFA	rs699947	0.34	0.916	0.046
VEGFA	rs13207351	0.357	0.952	0.035
VEGFA	rs1570360	0.459	0.69	0.05
VEGFA	rs3025030	0.731	0.451	0.884
VEGFA	rs3025039	0.378	0.184	0.592
VEGFA	rs10434	0.304	0.109	0.27
VEGFA	rs10738760	0.368	0.589	0.945
HFE	rs1800562	0.955	0.918	0.527
SLMAP	rs17058639	0.035	0.106	0.22
VDR	rs1544410	0.431	0.438	0.288
VDR	rs7975232	0.66	0.378	0.93
TGF-b1	rs1800470	0.015	0.226	0.02
ARHGAP22	rs4838605	0.065	0.691	0.048

## Data Availability

Once the project is complete, the data will be submitted to NIH GenBank https://www.ncbi.nlm.nih.gov/genbank.

## Abbreviations

ARHGAP22	Rho GTPase activating protein 22
C3	Complement component 3
C5	Complement component 5
CFB	Complement Factor B
CFH	Complement factor H
CFP	Color fundus photograph
DH	Deep hemorrhage
DM	Diabetes mellitus
DME	Diabetic macular edema
DNA	Deoxyribonucleic acid
DR	Diabetic retinopathy
DRSS	Diabetic retinopathy severity scale
ETDRS	Early treatment diabetic retinopathy study
FGF2	Fibroblast growth factor 2
HFE	Hemochromotosis
IRMA	Inraretinal microvascular abnormalities
IVT	Intravitreal
KDR	Kinase domain insert receptor
MA	Microaneurysm
NPDR	Non-proliferative diabetic retinopathy
PDR	Proliferative diabetic retinopathy
rs	Reference snp cluster ID
SLAMP	Sarcolemma associated protein
SNP	Single nucleotide polymorphism
STDR	Sight threatening diabetic retinopathy
T1DM	Type 1 diabetes mellitus
T2DM	Type 2 diabetes mellitus
UWF	Ultrawide field
VEGF	Vascular endothelial growth factor
VDR	Vitamin D receptor

## References

[1] Moss S.E., Klein R., Klein B.E. (1998). Ten-year Incidence of Visual Loss in a Diabetic Population. Ophthalmology.

[2] Klein B.E.K. (2007). Overview of Epidemiologic Studies of Diabetic Retinopathy. Ophthalmic Epidemiol..

[3] Cheung N., Mitchell P., Wong T.Y. (2010). Diabetic retinopathy. Lancet.

[4] Mohamed Q., Gillies M., Wong T.Y. (2007). Management of diabetic retinopathy: A systematic review. JAMA.

[5] Strowig S., Raskin P. (1992). Glycemic Control and Diabetic Complications. Diabetes Care.

[6] Simó-Servat O., Hernández C., Simó R. (2013). Genetics in diabetic retinopathy: Current concepts and new insights. Curr. Genom..

[7] Doria A. (2010). Genetics of Diabetes Complications. Curr. Diabetes Rep..

[8] Leslie R.D., Pyke D.A. (1982). Diabetic retinopathy in identical twins. Diabetes.

[9] Early Treatment Diabetic Retinopathy Study Research Group (1991). ETDRS report number 12, Fundus photographic risk factors for progression of diabetic retinopathy. Ophthalmology.

[10] Pearce E., Chong V., Sivaprasad S. (2020). Aflibercept Reduces Retinal Hemorrhages and Intravitreal Microvascular Abnormalities but Not Venous Beading: Secondary Analysis of the CLARITY Study. Ophthalmol. Retin..

[11] Broadgate S., Kiire C., Halford S., Chong V. (2018). Diabetic macular oedema: Under-represented in the genetic analysis of diabetic retinopathy. Acta Ophthalmol..

[12] Hampton B.M., Schwartz S.G., Brantley M.A., Flynn H.W. (2015). Update on genetics and diabetic retinopathy. Clin. Ophthalmol..

[13] Early Treatment Diabetic Retinopathy Study Research Group (1991). Grading diabetic retinopathy from stereoscopic color fundus photographs—An extension of the modified Airlie House classification. ETDRS report number 10. Ophthalmology.

[14] Price L., Au S., Chong V. (2015). Optomap ultrawide field imaging identifies additional retinal abnormalities in patients with diabetic retinopathy. Clin. Ophthalmol..

[15] Silva P.S., El-Rami H., Barham R., Gupta A., Fleming A., van Hemert J., Cavallerano J., Sun J.K., Aiello L.P. (2017). Hemorrhage and/or Microaneurysm Severity and Count in Ultrawide Field Images and Early Treatment Diabetic Retinopathy Study Photography. Ophthalmology.

[16] Walshe T.E., Saint-Geniez M., Maharaj A.S.R., Sekiyama E., Maldonado A.E., D’Amore P.A. (2009). TGF-β Is Required for Vascular Barrier Function, Endothelial Survival and Homeostasis of the Adult Microvasculature. PLoS ONE.

[17] Frank R.N. (2004). Diabetic retinopathy. N. Engl. J. Med..

[18] Duh E.J., Sun J., Stitt A.W. (2017). Diabetic retinopathy: Current understanding, mechanisms, and treatment strategies. JCI Insight.

[19] Buraczynska M., Baranowicz-Gaszczyk I., Borowicz E., Ksiazek A. (2007). TGF-beta1 and TSC-22 gene polymorphisms and susceptibility to microvascular complications in type 2 diabetes. Nephron Physiol..

[20] Liu L., Jiao J., Wang Y., Wu J., Huang D., Teng W., Chen L. (2014). TGF-beta1 Gene Polymorphism in Association with Diabetic Retinopathy Susceptibility: A Systematic Review and Meta-Analysis. PLoS ONE.

[21] Rodrigues K.F., Pietrani N.T., Sandrim V.C., Vieira C.M.A.F., Fernandes A.P., Bosco A.A., Gomes K.B. (2015). Association of a Large Panel of Cytokine Gene Polymorphisms with Complications and Comorbidities in Type 2 Diabetes Patients. J. Diabetes Res..

[22] Upadhyay R., Robay A., Fakhro K., Khadil C.A., Zirie M., Jayyousi A., El-Shafei M., Kiss S., D’Amico D.J., Salit J. (2015). Role of SLMAP genetic variants in susceptibility of diabetes and diabetic retinopathy in Qatari population. J. Transl. Med..

[23] Ding H., Howarth A.G., Pannirselvam M., Anderson T.J., Severson D.L., Wiehler W.B., Triggle C.R., Tuana B.S. (2005). Endothelial dysfunction in Type 2 diabetes correlates with deregulated expression of the tail-anchored membrane protein SLMAP. Am. J. Physiol.-Heart Circ. Physiol..

[24] Merle N.S., Church S., Fremeaux-Bacchi V., Roumenina L.T. (2015). Complement System Part I—Molecular Mechanisms of Activation and Regulation. Front. Immunol..

[25] Chrzanowska M., Modrzejewska A., Modrzejewska M. (2018). New insight into the role of the complement in the most common types of retinopathy-current literature review. Int. J. Ophthalmol..

[26] Wang J., Yang M., Li Y., Liu G., Teng Y., Liu X.M. (2013). Association of CFH and CFB gene polymorphisms with retinopathy in type 2 diabetic patients. Mediators Inflamm..

[27] Han J., Lando L., Skowronska-Krawczyk D., Chao D.L. (2019). Genetics of Diabetic Retinopathy. Curr. Diabetes Rep..

[28] Chen C.-F., Liou S.-W., Wu H.-H., Lin C.-H., Huang L.-S., Woung L.-C., Tsai C.-Y. (2016). Regulatory SNPs Alter the Gene Expression of Diabetic Retinopathy Associated Secretary Factors. Int. J. Med. Sci..

[29] Ray D., Mishra M., Ralph S., Read I., Davies R., Brenchley P. (2004). Association of the VEGF Gene With Proliferative Diabetic Retinopathy but Not Proteinuria in Diabetes. Diabetes.

[30] Churchill A.J., Carter J.G., Ramsden C., Turner S.J., Yeung A., Brenchley P.E.C., Ray D.W. (2008). VEGF Polymorphisms Are Associated with Severity of Diabetic Retinopathy. Investig. Ophthalmol. Vis. Sci..

[31] Simões M.J., Lobo C., Egas C., Nunes S., Carmona S., Costa M., Duarte T., Ribeiro L., Faro C., Cunha-Vaz J.G. (2014). Genetic Variants in ICAM1, PPARGC1A and MTHFR Are Potentially Associated with Different Phenotypes of Diabetic Retinopathy. Ophthalmologica.

[32] Stitt A.W., O’Neill C.L., O’Doherty M.T., Archer D.B., Gardiner T.A., Medina R.J. (2011). Vascular stem cells and ischaemic retinopathies. Prog. Retin. Eye Res..

[33] Chen L., Zhang X., Wen F. (2018). Venous beading in two or more quadrants might not be a sensitive grading criterion for severe nonproliferative diabetic retinopathy. Graefe’s Arch. Clin. Exp. Ophthalmol..

[34] Stitt A.W., Curtis T.M., Chen M., Medina R.J., McKay G.J., Jenkins A., Gardiner T.A., Lyons T.J., Hammes H.-P., Simó R. (2016). The progress in understanding and treatment of diabetic retinopathy. Prog. Retin. Eye Res..

[35] Imesch P.D., Bindley C., Wallow I.H. (1997). Clinicopathologic correlation of intraretinal microvascular abnormalities. Retina.

[36] Romero-Aroca P., Baget-Bernaldiz M., Fernandez-Ballart J., Plana-Gil N., Soler-Lluis N., Mendez-Marin I., Bautista-Perez A. (2011). Ten-year incidence of diabetic retinopathy and macular edema. Risk factors in a sample of people with type 1 diabetes. Diabetes Res. Clin. Pract..

[37] Thomas R.L., Dunstan F., Luzio S., Chowdury S.R., Hale S.L., North R., Gibbins R.L., Owens D.R. (2012). Incidence of diabetic retinopathy in people with type 2 diabetes mellitus attending the Diabetic Retinopathy Screening Service for Wales: Retrospective analysis. BMJ.

[38] Jones C.D., Greenwood R.H., Misra A., Bachmann M.O. (2012). Incidence and Progression of Diabetic Retinopathy During 17 Years of a Population-Based Screening Program in England. Diabetes Care.

[39] Xu J., Xu L., Wang Y., You Q., Jonas J., Wei W.B. (2014). Ten-year cumulative incidence of diabetic retinopathy. The Beijing Eye Study 2001/2011. PLoS ONE.

